# Cryopreserved mesenchymal stromal cells display impaired immunosuppressive properties as a result of heat-shock response and impaired interferon-γ licensing

**DOI:** 10.3109/14653249.2011.623691

**Published:** 2011-10-27

**Authors:** Moïra François, Ian B Copland, Shala Yuan, Raphaëlle Romieu-Mourez, Edmund K Waller, Jacques Galipeau

**Affiliations:** 1 Department of Experimental Medicine, McGill University, Montréal, Canada; 2 Lady Sir Mortimer B. Davis Jewish General Hospital and Lady Davis Institute for Medical Research, Montréal, Canada; 3 Department of Hematology and Medical Oncology, Emory University Winship Cancer Institute, Atlanta, Georgia, USA; 4 Department of Pediatrics, Emory University Winship Cancer Institute, Atlanta, Georgia, USA

**Keywords:** cryopreservation, immunosuppression, immunotherapy, indoleamine 2,3-dioxygenase, interferon-gamma, mesenchymal stromal cells

## Abstract

Human mesenchymal stromal cells (MSC) can suppress T-cell activation *in vitro* in an indoleamine 2,3-dioxygenase (IDO)-dependent manner. However, their clinical effects on immune ailments have been inconsistent, with a recent phase III study showing no benefit in acute graft-versus-host disease (GvHD). We here tested the hypothesis that the banked, cryopreserved MSC often used in clinical trials display biologic properties distinct from that of MSC in the log phase of growth typically examined in pre-clinical studies. In freshly thawed cryopreserved MSC derived from normal human volunteers, we observed that MSC up-regulate heat-shock proteins, are refractory to interferon (IFN)-γ-induced up-regulation of IDO, and are compromised in suppressing CD3/CD28-driven T cell proliferation. Immune suppressor activity, IFN-γ responsiveness and induction of IDO were fully restored following 24 h of MSC tissue culture post-thaw. These results highlight a possible cause for the inefficacy of MSC-based immunotherapy reported in clinical trials using cryopreserved MSC thawed immediately prior to infusion.

## Introduction

One mechanism by which human bone marrow-derived mesenchymal stromal cells (MSC) suppress T-cell proliferation *in vitro* is mediated by the induction of indoleamine 2,3-dioxygenase (IDO) (1,2). Because of their ability to inhibit activated T cells specifically, MSC have been the subject of phase I/II trials for their potential use as a cell-based immunotherapeutic for the treatment of autoimmune and alloimmune disorders (3-6). Clinical studies have demonstrated that the administration of autologous or allogeneic MSC is safe, and numerous phase II studies have suggested encouraging potency, particularly in the treatment of steroid-resistant graft-versus-host disease (GvHD) (7). However, therapeutic effectiveness of MSC is not universally observed (6), including the results of a large phase III trial of major histocompatibility complex (MHC)-unmatched allogeneic MSC for GvHD recently completed in the USA (8).

The current model for MSC-based immunotherapy is to expand cells *ex vivo*, cryogenically bank them until needed, then administer thawed MSC intravenously to the patient. Until now, the effect of cryopreservation recovery on the immunosuppressive properties of MSC has not been investigated. With the present study, we demonstrate that freshly thawed MSC are unable to suppress T-cell proliferation *in vitro*, which correlates with impaired up-regulation of IDO in response to interferon (IFN)-γ. These observations provide some insight on the inconsistencies between the pre-clinical and clinical utility of MSC for treatment of immune ailments.

## Methods

### Cells

Human MSC were isolated and cultured as described elsewhere (9). After the third passage, the MSC cultures were assayed by flow cytometry analysis for the absence of CD45^+^ and CD31 ^+^ contaminating cells and expression of CD44, CD73, CD90 and CD 105 (BD Biosdence, Mississauga, ON, Canada), and subjected to a differentiation assay toward adipocyte and osteocyte lineages, as described elsewhere (10). Human peripheral blood mononuclear cells (PBMC) were isolated from leukapheresis obtained from healthy volunteers using a Ficoll density gradient.

### Cell viability

Frozen MSC were thawed, washed in media and cultured for 4 h, 24 h or 7 days before all cells, floating and adherent, were analyzed using an annexin V and propidium iodide (PI) staining kit (Invitrogen, Burlington, ON, Canada) and quantified by flow cytometry. Trypan blue exclusion staining was also carried out in parallel, and a consistently greater ‘viability’ assessment was observed compared with annexinV/PI staining (trypan blue viability >80%, annexinV/PI viability ≤ 60%, immediately after thawing). We believe this is due to the fact that trypan blue staining only shows dead cells and does not include dying cells; therefore trypan blue values were disregarded.

### T cell proliferation assay

Carboxyfluorescein succinimidyl ester (CFSE; Invitrogen)-labeled PBMC were cultured at 400 000 cells/well in a 24-well plate with cultured, freshly thawed or paraformaldehyde (PFA)-fixed MSC at a ratio of MSC:PBMC of 1:3 and 1:9. T lymphocytes were stimulated using 0.4 μg/mL anti-human CD3 and CD28 antibodies (clone HIT3a and CD28.2, respectively, from BD Pharmagen, Mississauga, ON, Canada). T-cell proliferation was determined 4 days later by flow cytometry analysis of CFSE fluorescence intensity.

### Western blot

Anti-human IDO (GenScript, Piscataway, NJ, USA) and anti-human phospho-STAT-1 (clone 58D6; Cell Signaling, Pickering, ON, Canada) -specific antibodies were used to detect protein expression on whole cell protein extracts isolated from MSC by immunoblot analysis. Where indicated, MSC were activated with recombinant human IFN-γ (5 ng/mL; R&D System, Burlington, ON, Canada).

### Real-time quantitative polymerase chain reaction

DNA-free total RNA was extracted and reverse transcribed as described elsewhere (10). Real-time quantitative polymerase chain reaction (qPCR) assays were performed in duplicate on an ABI 7500 fast real-time PCR system thermal cycler and SYBR green mastermix (Applied Biosystems, Carlsbad, CA, USA) with human primer sequences (5'3’ forward, reverse) for: IDO, GCCCTTCAAGTGTTTCACCAA, CCAGCC AGACAAATATATGCGA; Chemokine (C-C motif) ligand 2 (CCL2), CAAGCAGAAGTGGGTTCAG-GAT, TCTTCGGAGTTTGGGTTTGC; interleukin (IL-6), ATGAAGTTCCTCTCTGCAAGAGACT, CACTAGGTTTGCCGAGTAGATCTQheatshock protein (Hsp27), CTGGCGCGTGTCCCTGGATG, GCTAGCTTGGGCATGGGGGC; Hsp47, CTGC TAGTCAACGCCATGTTCTTC, TCTGTGTCCA ACTCAAAGGQ Hsp56, TCCTCACCCCCGACG-GTGTG, CTGGCTTGTGCTGCCTGGCT; Hsp 70A,GGTCCGGATAACGGCTAGCCT,CCACCG GGTCGCCGAACTTG; Hsp70B, CATGCCCCG ATCTGCCCGAAC, GCACCTTCCCGCCCAGT TGAG; Hsp90, ATGGCTGCTTCCCAGGTGAT GCC, ACCTCAGGCTCATGACACCAGC; 18S ribosomal RNA (18S), TTACCAAAAGTGGCCCA CTA, GAAAGATGGTGAACTATGCC. Primers were designed using the National Center for Biotechnology Information (NCBI)/primer blast designing tool. Data were analyzed using the relative quantification method (11).

### Statistical analysis

Data are reported as mean ± standard deviation (SD). All calculations were carried out using Graph-Pad Prism software (GraphPad Software, La Jolla, CA, USA). Comparisons between groups were made by anova.

## Results and discussion

**Figure 1:**
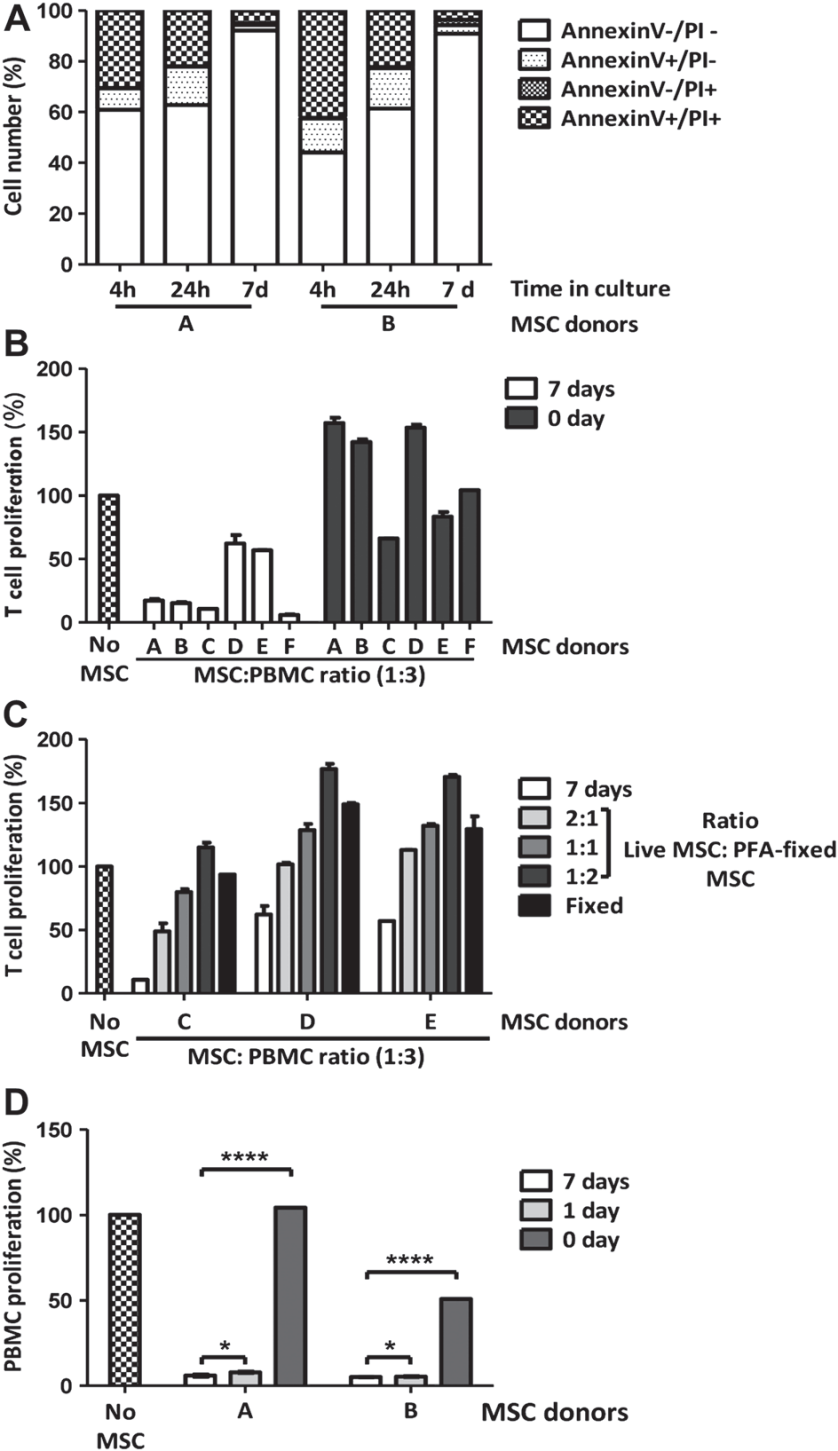
Immunosuppressive potential of freshly thawed human MSC compared with MSC in culture. (A) Cell viability analysis was performed on freshly thawed and cultured MSC using annexin V and PI labeling. (B) T-cell proliferation assays were performed using CFSE-labeled human PBMC activated with 0.4 μg/mL anti-CD3 and -CD28 antibodies and co-cultured for 4 days with or without MSC maintained in culture for 7 days or freshly thawed at a MSC:PBMC ratio of 1:3. Cell proliferation was determined by flow cytometry after gating lymphocytes on the forward and side scatter plot and measuring the percentage of CFSE^low^ T cells. (C) T-cell proliferation assay performed as in (B) using cultured MSC mixed with PFA-fixed MSC at a ratio of live:fixed of 2:1, 1:1, 1:2 or 100% fixed. A MSC:PBMC ratio of 1:3 was used. (D) T-cell proliferation assays performed as in (B) on two MSC donors freshly thawed and cultured for 1 and 7 days. Figures show representative results with means ± SD.

To determine the immunosuppressive potential of cryopreserved MSC toward T-cell activation, we analyzed their potency to suppress PBMC proliferation in comparison with MSC maintained in culture for 7 days. Early passage MSC were frozen in α Minimum Essential Medium (MEM), 30% fetal bovine serum and 5% dimethyl sulfoxide at −80°C for 24 h, then transferred to liquid nitrogen for 1 week before use. Cell viability analysis by flow cytometry using annexin V- and PI-labeling revealed that, in cultured MSC, the percentage of live cells (annexin V^−^ and PI^"^) was 92% and 91% for donors A and B, respectively, while in their thawed counterparts the percentage of live cells dropped to 61% and 44%, respectively (Figure 1A). We next tested post-thaw cell culture restoration of immune suppressor function. Freshly thawed cryopreserved MSC and MSC in the log phase of growth were co-cultured with CFSE-labeled PBMC activated with anti-CD3 and -CD28 antibodies at a MSC:PBMC ratio of 1:3, and T-cell proliferation was measured 4 days later. Cryopreserved MSC had a significantly impaired inhibitory effect on the proliferation of activated T cells in comparison with their cultured counterpart maintained in the log phase of growth (*P*< 0.0001) (Figure 1B). T-cell hyperproliferation observed in the presence of thawed MSC suggested that dead MSC found in the freshly thawed MSC samples may activate T-cell proliferation. To test this hypothesis, we performed a T-cell proliferation assay in which cultured MSC were mixed at a different ratio with PFA-fixed MSC, which served as dead cells, while the MSC:PBMC ratio remained 1:3 (Figure 1C). Adding increasing amounts of fixed MSC while reducing the amount of live MSC diminished the level of T-cell immunosuppression. Hyper-proliferation of T cells could also be observed at ratios of 1:1, 1:2 and 100% fixed, depending on the MSC donor, which may have been caused by the low amount of IDO produced in comparison with the pro-inflammatory cytokines [i.e. interleukin (IL)-6] produced by live MSC. Four hours of adherent or suspension culture of MSC after thawing were not sufficient to restore their immunosuppressive capabilities (data not shown). However, a culture period of 24 h was sufficient to restore the immunosuppressive properties of thawed MSC to a level similar to that of MSC cultured for 7 days (Figure 1D).

**Figure 2:**
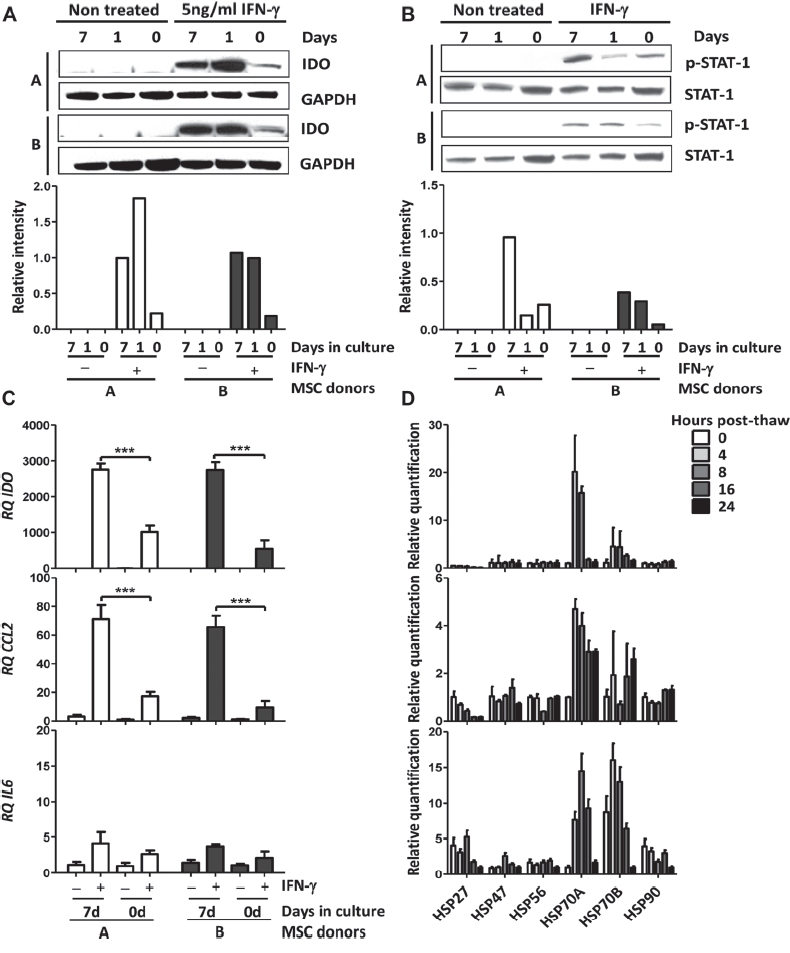
Protein and mRNA expression level of freshly thawed human MSC compared with MSC in culture. (A) IDO protein expression was analyzed by immunoblot on two human MSC donors freshly thawed and cultured for 1 and 7 days. IDO protein expression was induced by stimulating the MSC with 5 ng/mL recombinant (rh)IFN-γ for 24 h. Protein expression of Glyceraldehyde-3-phosphate dehydrogenase (GAPDH) was used as a loading control. (B) Phosphorylated STAT-1 protein expression was analyzed by immunoblot on two human MSC donors freshly thawed and cultured for 1 and 7 days. STAT-1 phosphorylation was induced by stimulating the MSC with 5 ng/mL IFN-γ for 20 min. Protein expression of total STAT-1 was used as a loading control. (C) mRNA expression levels of (i) IDO, (ii) CCL2 and (iii) IL6 were measured by real-time qPCR in two MSC donors freshly thawed and cultured for 7 days. MSC were left untreated or stimulated with 5 ng/mL rhIFN-γ for 24 h. (D) mRNA expression level of Hsp27, Hsp47, Hsp56, Hsp70A, Hsp70B and Hsp90 by real-time qPCR performed on three MSC donors immediately after thawing and following a post-thaw recovery period of 4, 8, 16 and 24 h. Ribosomal 18S RNA was used as an internal control. Figures show representative results with means ± SD.

IFN-γ-inducible expression of IDO in human MSC has been identified as one of the main mechanisms of T-cell immunosuppression (2). We therefore tested the induction of IDO protein expression in MSC. Freshly thawed MSC stimulated with IFN-γ showed very low levels of IDO protein expression compared with cultured MSC, while culture recovery for at least 24 h restored IFN-γ responsiveness and IDO induction (Figure 2A). This observation suggests that the MSC biochemical response to IFN-γ and/or the protein synthesis machinery is compromised. To test this hypothesis, we analyzed Signal Transducers and Activators of Transcription (STAT-1) phosphorylation in response to IFN-γ stimulation by immunoblotting, and found that phosphorylated STAT-1 was markedly diminished in thawed compared with cultured MSC (Figure 2B). Consistent with these data, we queried the mRNA transcript levels of IDO, and of two constitutively expressed genes, CCL2 and IL-6. No basal expression mRNA encoding of IDO was detected, while the basal levels of CCL2 and IL-6 mRNA gene expression were comparable in freshly thawed and cultured MSC (Figure 2C). In response to IFN-γ, the up-regulation of IDO and CCL2 mRNA expression was significantly diminished in thawed MSC. In two individual donors, IFN-γ-induced levels of IDO mRNA decreased 2.7- and 5-fold (*P*< 0.001; Figure 2Ci), while CCL2 mRNA expression decreased 4- and 7-fold (*P*<0.001; Figure 2Cii). IL-6 regulation is mainly regulated by tumor necrosis factor (TNF)-α, which appeared only slightly affected by IFN-γ stimulation (Figure 2Ciii). These results suggest that transcript levels of constitutively produced proteins are not altered while transcription of IFN-γ-inducible genes is. Studies performed on human fibroblasts have demonstrated that cryopreservation and thawing induces the expression of heat-shock proteins (Hsp) Hsp27, Hsp47, Hsp56 and Hsp90, which diminishes within 24 h (12). In addition, Hsp27 has been demonstrated to inhibit cellular protein synthesis by blocking the assembly of the translation cap initiation complex (13). In order to determine whether the transcription of Hsp genes is altered by stress induced by the cryopreservation and thawing process in MSC, we performed real-time qPCR on MSC samples immediately after thawing and following a post-thaw culture recovery period of 4,8, 16 and 24 h. For this assay, primer sequences for Hsp27, Hsp47, Hsp56, Hsp70A, Hsp70B and Hsp90 were tested. We observed an up-regulation of mRNA level of Hsp70A and Hsp70B as early as 4 h post-thaw, which peaked at 8 h post-thaw and returned to basal levels within 24 h for three individual MSC donors tested (Figure 2D). These data support the theory that thawed MSC recapitulate a molecular genetic heat-shock response that reverses within 24 h, and provides a mechanistic rationale for the loss of IFN-γ responsiveness and blunted suppressive licensing (14). Therefore, we speculate that the stress response induced by cryopreservation recovery may temporarily prevent MSC from responding to immune cues and secondary suppressor functions.

In conclusion, our data demonstrate that cryopreservation negatively affects the immunosuppressive properties of MSC in a reversible manner, and is associated with a heat-shock stress response initiated during the thawing process. A culture recovery period of at least 24 h is able to restore the immunosuppressive properties of MSC, including transcriptional IDO responsiveness to IFN-γ and down-regulation of Hsp expression. In addition, phosphatidylserine translocation to the cell surface is a reversible process and not a strict commitment to apoptosis (15,16). Temporary repression of non-constitutively expressed genes during the stress response allows the cells to prioritize cell survival before recovering their functional properties (14). Allowing MSC to recover for a period as short as 24 h post-thaw may reverse early apoptosis, initiate internalization of phosphatidylserine, and possibly prevent *in vivo* clearance of annexin V-positive MSC by phagocytes (17,18), in addition to down-regulating the cellular stress response. We propose that the potency of MSC as a suppressor therapy for auto/alloimmune ailments may be improved if delivered in a metabolically optimal condition.
